# Interim Estimates of 2024–2025 Seasonal Influenza Vaccine Effectiveness — Four Vaccine Effectiveness Networks, United States, October 2024–February 2025

**DOI:** 10.15585/mmwr.mm7406a2

**Published:** 2025-02-27

**Authors:** Aaron M. Frutos, Seana Cleary, Emily L. Reeves, Haris M. Ahmad, Ashley M. Price, Wesley H. Self, Yuwei Zhu, Basmah Safdar, Ithan D. Peltan, Kevin W. Gibbs, Matthew C. Exline, Adam S. Lauring, Sarah W. Ball, Malini DeSilva, Sara Y. Tartof, Kristin Dascomb, Stephanie A. Irving, Nicola P. Klein, Brian E. Dixon, Toan C. Ong, Ivana A. Vaughn, Stacey L. House, Kiran A. Faryar, Mary Patricia Nowalk, Manjusha Gaglani, Karen J. Wernli, Vel Murugan, Olivia L. Williams, Rangaraj Selvarangan, Geoffrey A. Weinberg, Mary A. Staat, Natasha B. Halasa, Leila C. Sahni, Marian G. Michaels, Janet A. Englund, Marie K. Kirby, Diya Surie, Fatimah S. Dawood, Benjamin R. Clopper, Heidi L. Moline, Ruth Link-Gelles, Amanda B. Payne, Elizabeth Harker, Kristina Wielgosz, Zachary A. Weber, Duck-Hye Yang, Nathaniel M. Lewis, Jennifer DeCuir, Samantha M. Olson, Jessie R. Chung, Brendan Flannery, Lisa A. Grohskopf, Carrie Reed, Shikha Garg, Sascha Ellington, CDC Influenza Vaccine Effectiveness Collaborators, CDC Influenza Vaccine Effectiveness Collaborators, Laurence W. Busse, Cristie Columbus, Abhijit Duggal, Adit A. Ginde, Michelle N. Gong, David N. Hager, Estelle Harris, Cassandra Johnson, Nicholas J. Johnson, Akram Khan, Jennie H. Kwon, Christopher Mallow, Nicholas M. Mohr, Jarrod M. Mosier, Matthew E. Prekker, Nida Qadir, Colleen Ratcliff, Nathan I. Shapiro, Jay S. Steingrub, Jennifer G. Wilson, Omobosola Akinsete, Michelle Barron, Daniel Bride, Tom Duszynski, Shaun Grannis, John Hansen, Padma Koppolu, David Mayer, Charlene McEvoy, Allison L. Naleway, S. Bianca Salas, Tamara Sheffield, Lina S. Sy, Ousseny Zerbo, Julie A. Boom, Megan Freeman, Eileen J. Klein, Mary E. Moffatt, Daniel C. Payne, Pedro A. Piedra, Elizabeth P. Schlaudecker, Jennifer E. Schuster, Laura S. Stewart, Peter G. Szilagyi, John V. Williams, Danielle M. Zerr, G.K. Balasubramani, Natalie A. B. Bontrager, Tara Curley, Curtis Donskey, Juliana DaSilva, Britan Fairall, Krissy Moehling Geffel, Claudia Hoyen, Lisa M. Keong, Erika Kiniry, Aleda M. Leis, Emily T. Martin, Jamie Mills, Lora Nordstrom, Leah Odame-Bamfo, C. Hallie Phillips, Emmanuel B. Walter, Karen Yeager

**Affiliations:** ^1^Influenza Division, National Center for Immunization and Respiratory Diseases, CDC; ^2^Epidemic Intelligence Service, CDC; ^3^Vanderbilt University Medical Center, Nashville, Tennessee; ^4^Yale University, New Haven, Connecticut; ^5^Intermountain Medical Center, Salt Lake City, Utah; ^6^Wake Forest University School of Medicine, Winston-Salem, North Carolina; ^7^The Ohio State University Wexner Medical Center, Columbus, Ohio; ^8^University of Michigan School of Medicine, Ann Arbor, Michigan; ^9^Clinical Research Practice, Westat, Rockville, Maryland; ^10^HealthPartners Institute, Minneapolis, Minnesota; ^11^Department of Research & Evaluation, Kaiser Permanente Southern California, Pasadena, California; ^12^Division of Infectious Diseases and Clinical Epidemiology, Intermountain Health, Salt Lake City, Utah; ^13^Science Programs Department, Kaiser Permanente Center for Health Research, Portland, Oregon; ^14^Kaiser Permanente Vaccine Study Center, Kaiser Permanente Northern California Division of Research, Oakland, California; ^15^Center for Biomedical Informatics, Regenstrief Institute, Indianapolis, Indiana; ^16^Fairbanks School of Public Health, Indiana University, Indianapolis, Indiana; ^17^Department of Biomedical Informatics, University of Colorado Anschutz Medical Campus, Aurora, Colorado; ^18^Department of Public Health Sciences, Henry Ford Health, Detroit, Michigan; ^19^Department of Emergency Medicine, Washington University School of Medicine, St. Louis, Missouri; ^20^Department of Emergency Medicine, University Hospitals of Cleveland, Cleveland, Ohio; ^21^Department of Family Medicine, University of Pittsburgh, Pittsburgh, Pennsylvania; ^22^Department of Pediatrics, Baylor Scott & White Health, Temple, Texas; ^23^Department of Pediatrics, Baylor College of Medicine, Temple, Texas; ^24^Kaiser Permanente Washington Health Research Institute, Seattle, Washington; ^25^Kaiser Permanente Bernard J. Tyson School of Medicine, Pasadena, California; ^26^Biodesign Center for Personalized Diagnostics, Arizona State University, Tempe, Arizona; ^27^Duke Human Vaccine Institute, Duke University School of Medicine, Durham, North Carolina; ^28^University of Missouri-Kansas City School of Medicine, Kansas City, Missouri; ^29^Children’s Mercy Hospital, Kansas City, Missouri; ^30^University of Rochester School of Medicine and Dentistry, Rochester, New York; ^31^Cincinnati Children’s Hospital Medical Center and University of Cincinnati College of Medicine, Cincinnati, Ohio; ^32^Texas Children’s Hospital and Baylor College of Medicine, Houston, Texas; ^33^UPMC Children’s Hospital of Pittsburgh, University of Pittsburgh School of Medicine, Pittsburgh, Pennsylvania; ^34^Seattle Children’s Research Institute, Seattle, Washington; ^35^Coronavirus and Other Respiratory Viruses Division, National Center for Immunization and Respiratory Diseases, CDC.; Emory University; Baylor University Medical Center; Cleveland Clinic; University of Colorado; Montefiore Medical Center; Johns Hopkins University; University of Utah; Vanderbilt University Medical Center; University of Washington; Oregon Health & Science University; Washington University; University of Miami; University of Iowa; University of Arizona; Hennepin County Medical Center; University of California, Los Angeles; Vanderbilt University Medical Center; Beth Israel Deaconess Medical Center; Baystate Medical Center; Stanford University; HealthPartners Institute; University of Colorado Anschutz Medical Campus; Intermountain Health; Indiana University; Regenstrief Institute; Kaiser Permanente Northern California; Kaiser Permanente Center for Health Research; University of Colorado Anschutz Medical Campus; HealthPartners Institute; Kaiser Permanente Center for Health Research; Kaiser Permanente Southern California; Intermountain Health; Kaiser Permanente Southern California; Kaiser Permanente Northern California; Baylor College of Medicine and Texas Children’s Hospital; University of Pittsburgh School of Medicine; Seattle Children’s Research Institute; Children’s Mercy Hospital and University of Missouri-Kansas City School of Medicine; Cincinnati Children’s Hospital Medical Center and University of Cincinnati College of Medicine; Baylor College of Medicine and Texas Children’s Hospital; Cincinnati Children’s Hospital Medical Center and University of Cincinnati College of Medicine; Children’s Mercy Hospital and University of Missouri-Kansas City School of Medicine; Vanderbilt University Medical Center; University of Rochester School of Medicine and Dentistry and UCLA Mattel Children's Hospital; UPMC Children’s Hospital of Pittsburgh; University of Pittsburgh School of Medicine; and University of Wisconsin School of Medicine and Public Health; Seattle Children’s Research Institute; University of Pittsburgh; Duke Human Vaccine Institute; Washington University School of Medicine in St. Louis; Louis Stokes Cleveland Veterans Administration Medical Center; Influenza Division, National Center for Immunization and Respiratory Diseases, CDC; Baylor Scott & White Research Institute; University of Pittsburgh; University of Cleveland Hospitals Rainbow Babies & Children’s Hospital; Influenza Division, National Center for Immunization and Respiratory Diseases, CDC; Kaiser Permanente Washington Health Research Institute; University of Michigan; University of Michigan; Washington University School of Medicine in St. Louis; Valleywise Health Medical Center; Baylor Scott & White Research Institute; Kaiser Permanente Washington Health Research Institute; Duke Human Vaccine Institute; Phoenix Children’s Hospital

**Summary**

**What is already known about this topic?**

CDC routinely monitors influenza vaccine effectiveness (VE). Annual influenza vaccination is recommended for all eligible persons aged ≥6 months.

**What is added by this report?**

Interim 2024–2025 seasonal influenza VE estimates were derived from four U.S. VE networks. Among children and adolescents, VE was 32%, 59%, and 60% in outpatient settings (three networks) and 63% and 78% against influenza-associated hospitalization (two networks). Among adults, VE was 36% and 54% in outpatient settings (two networks) and 41% and 55% against influenza-associated hospitalization (two networks).

**What are the implications for public health practice?**

Vaccination with the 2024–2025 influenza vaccine reduced the risk for influenza-associated outpatient visits and hospitalization. These findings support recommendations that all eligible persons aged ≥6 months should receive an annual influenza vaccination. Vaccination should be offered as long as influenza viruses are circulating.

## Abstract

Annual influenza vaccination is recommended for all persons aged ≥6 months in the United States. Interim influenza vaccine effectiveness (VE) was calculated among patients with acute respiratory illness–associated outpatient visits and hospitalizations from four VE networks during the 2024–25 influenza season (October 2024–February 2025). Among children and adolescents aged <18 years, VE against any influenza was 32%, 59%, and 60% in the outpatient setting in three networks, and against influenza-associated hospitalization was 63% and 78% in two networks. Among adults aged ≥18 years, VE in the outpatient setting was 36% and 54% in two networks and was 41% and 55% against hospitalization in two networks. Preliminary estimates indicate that receipt of the 2024–2025 influenza vaccine reduced the likelihood of medically attended influenza and influenza-associated hospitalization. CDC recommends annual receipt of an age-appropriate influenza vaccine by all eligible persons aged ≥6 months as long as influenza viruses continue to circulate locally.

## Introduction

Because of continual evolutionary changes in influenza viruses, CDC regularly monitors* influenza vaccine effectiveness (VE). Influenza vaccination prevents hundreds of thousands of outpatient medical visits, tens of thousands of hospitalizations, and thousands of deaths from influenza every year.^†^ CDC’s Advisory Committee on Immunization Practices recommends annual seasonal influenza vaccination for all persons aged ≥6 months (*1*). In March 2024, after the absence of detections of influenza B/Yamagata lineage viruses since 2020,^§^ the Food and Drug Administration recommended changing from a quadrivalent vaccine (including four influenza virus antigens) to a trivalent vaccine, containing three influenza virus antigens. During the 2024–25 influenza season, most influenza viruses detected in the United States were influenza A viruses (97% of positive specimens); among subtyped influenza A–positive specimens, 52% were influenza A(H3N2), and 47% were A(H1N1)pdm09 viruses.^¶^ This report provides interim estimates of effectiveness of any 2024–2025 influenza vaccine (i.e., trivalent inactivated influenza vaccine, trivalent recombinant influenza vaccine, or trivalent live attenuated influenza vaccine) against medically attended, laboratory-confirmed influenza for persons in the outpatient and inpatient settings from four U.S. VE surveillance networks.

## Methods

### Data Source and Collection

Analyses were conducted using data from four CDC-affiliated VE networks, all of which use a test-negative, case-control design to evaluate influenza VE: 1) Investigating Respiratory Viruses in the Acutely Ill (IVY), 2) the New Vaccine Surveillance Network (NVSN), 3) U.S. Flu Vaccine Effectiveness (U.S. Flu VE), and 4) the Virtual SARS-CoV-2, Influenza, and Other respiratory viruses Network (VISION). These analyses include child and adolescent and adult patients who received medical care (outpatient or inpatient) for an acute respiratory illness (ARI) during the 2024–25 influenza season. Case-patients were those persons with ARI who received a positive influenza molecular assay test result,** and control patients were those with ARI who received a negative influenza molecular assay test result.

The setting and age of enrolled patients differed by network (Box). IVY enrolled hospitalized patients aged ≥18 years. NVSN enrolled child and adolescent patients (aged 6 months–17 years) in the outpatient setting,^††^ as well as those admitted to a hospital. U.S. Flu VE enrolled child and adolescent (aged 8 months–17 years) and adult (≥18 years) patients in the outpatient setting. VISION included child and adolescent (aged 6 months–17 years) and adult (aged ≥18 years) patients in the outpatient setting and those admitted to a hospital.

BOX **Characteristics of four influenza vaccine effectiveness networks — United States, 2024–25 influenza season**

**1. Investigating Respiratory Viruses in the Acutely Ill Network**

- **Population:** adults aged ≥18 years
- **Setting**: inpatient only
- **Inclusion dates**: October 1, 2024–February 4, 2025
- **Type of surveillance**: active
- **Medical centers included (state)**: Baylor Scott & White Medical Center - Temple (Texas), Baylor Scott & White - Baylor University Medical Center (Texas), Baystate Medical Center (Massachusetts), Beth Israel Deaconess Medical Center (Massachusetts), Cleveland Clinic (Ohio), Emory University Medical Center (Georgia), Hennepin County Med. Ctr. (Minnesota), Henry Ford Health (Michigan), Intermountain Medical Center (Utah), Johns Hopkins Hospital (Maryland), Montefiore Medical Center (New York), The Ohio State University Wexner Medical Center (Ohio), Oregon Health and Science University Hospital (Oregon), Stanford University Medical Center (California), University of California, Los Angeles Medical Center (California), University of Colorado Hospital (Colorado), University of Iowa Hospitals (Iowa), University of Miami Medical Center (Florida), University of Michigan Hospital (Michigan), University of Utah (Utah), University of Washington (Washington), Vanderbilt University Medical Center (Tennessee), Wake Forest University Baptist Medical Center (North Carolina), Barnes-Jewish Hospital (Missouri), University of Arizona Medical Center (Arizona), and Yale University (Connecticut)
- **Determination of vaccination status**: influenza vaccination status was ascertained using jurisdictional immunization registries, electronic medical records, and by plausible patient or proxy report in the absence of source documentation
- **ARI definition**: one or more of the following: fever, cough, shortness of breath, new hypoxemia, or new pulmonary findings on chest imaging consistent with pneumonia
- **Influenza A subtype available**: yes

**2. New Vaccine Surveillance Network**

- **Population:** children and adolescents aged 6 months–17 years
- **Settings**: outpatient (outpatient clinics, urgent care clinics, and emergency departments); inpatient
- **Inclusion dates**: October 2, 2024–January 30, 2025
- **Type of surveillance**: primarily active*
- **Medical centers included (state)**: Vanderbilt University Medical Center (Tennessee), University of Rochester Medical Center (New York), Cincinnati Children’s Hospital Medical Center (Ohio), Texas Children’s Hospital (Texas), Seattle Children’s Hospital (Washington), Children’s Mercy Hospital (Missouri), and University of Pittsburgh Medical Center Children’s Hospital of Pittsburgh (Pennsylvania)
- **Determination of vaccination status**: jurisdictional immunization registries, medical records or self-report
- **ARI definition**: symptoms of acute respiratory illness (including cough, fever, or other symptoms) within 10 days of illness onset
- **Influenza A subtype available**: Yes

**3. United States Flu Vaccine Effectiveness Network**

- **Population:** children and adolescents aged 8 months–17 years; adults aged ≥18 years
- **Settings**: outpatient (outpatient clinics, urgent care clinics, and emergency departments)
- **Inclusion Dates**: October 1, 2024–January 17, 2025
- **Type of surveillance**: active
- **Medical centers included (state)**: Arizona State University Tempe, Phoenix Children’s Hospital, Valleywise Health Medical Center (Arizona), University of Michigan and Henry Ford Health (Michigan), Washington University in St. Louis (Missouri), University Hospitals of Cleveland and Louis Stokes Cleveland Department of Veterans Affairs Medical Center (Ohio), University of Pittsburgh, University of Pittsburgh Medical Center (Pennsylvania), Baylor Scott & White Health (Texas), and Kaiser Permanente Washington (Washington)
- **Determination of vaccination status**: medical records/jurisdictional immunization registries and self-report
- **ARI definition**: illness ≤7 days duration with new or worsening cough
- **Influenza A subtype available**: yes

**4. Virtual SARS-CoV-2, Influenza and Other respiratory viruses Network**

- **Population:** children and adolescents aged 6 months–17 years; adults aged ≥18 years
- **Settings**: outpatient (urgent care clinics and emergency departments); inpatient
- **Inclusion dates**: October 1, 2024–January 24, 2025
- **Type of surveillance**: passive
- **Medical centers included (state)**: HealthPartners (Minnesota and Wisconsin), Intermountain Health (Utah), Kaiser Permanente Northern California (California), Kaiser Permanente Southern California (California); Kaiser Permanente Center for Health Research (Oregon and Washington); Regenstrief Institute (Indiana); and UCHealth (Colorado)
- **Determination of vaccination status**: jurisdictional immunization registries, electronic health records, and claims data
- **ARI definition**: acute respiratory clinical diagnoses or respiratory signs or symptoms based on ICD-10 codes
- **Influenza A subtype available**: no

**Abbreviations:** ARI = acute respiratory illness; ICD-10 = *International Classification of Diseases, Tenth Revision*.

* For this analysis, 94% of New Vaccine Surveillance Network patients were enrolled through active surveillance.

### Data Analysis

To assess effects of vaccination on likelihood of influenza illness, VE was estimated as (1 − adjusted odds ratio) × 100% using multivariable logistic regression, adjusting for geographic region, age, calendar time of illness, and other prespecified confounders.^§§^ Patients were considered to be vaccinated if they received ≥1 dose of the 2024–2025 seasonal influenza vaccine ≥14 days before the date of ARI onset or clinical encounter.^¶¶^ Patients were excluded*** if they were vaccinated <14 days before the index date or had received a positive SARS-CoV-2 molecular assay test result (*2*). IVY, NVSN, and U.S. Flu VE calculated VE against influenza A virus subtypes A(H1N1)pdm09 and A(H3N2), when possible. VE point estimates for each network are reported, with 95% CIs included in the tables of this report; 95% CIs that exclude zero were considered statistically significant. For each network and patient age group, VE and 95% CIs were interpreted as the percentage of specific influenza outcomes prevented. SAS software (version 9.4; SAS Institute) and R (version 4.4; R Foundation) were used to conduct the analyses. IVY, NVSN, and U.S. Flu VE activities were reviewed by CDC, deemed not research, and were conducted consistent with applicable federal law and CDC policy.^†††^ VISION activities were reviewed by CDC and conducted consistent with applicable federal law and CDC policy.^§§§^

## Results

Data from the IVY network included 3,175 hospitalized adult patients aged ≥18 years with ARI (Table 1) (Supplementary Table 1, https://stacks.cdc.gov/view/cdc/176587). NVSN included 4,611 patients aged <18 years with ARI, including 2,969 seen in outpatient settings and 1,642 who were hospitalized. Among 3,344 patients with ARI in the outpatient setting included in the U.S. Flu VE network, 1,134 were patients aged <18 years, and 2,210 were adults. VISION data included 139,558 outpatient encounters (36,919 among patients aged <18 years and 102,639 among adults) and 32,671 hospitalized encounters (1,638 among patients aged <18 years and 31,033 among adults).

**TABLE 1:** Number and percentage of patients who received medical care for an acute respiratory illness, by medical care setting, age group, and influenza test result — four vaccine effectiveness networks, United States, 2024–25 influenza season

Network/Patient age group	Outpatient setting*^,†^	Influenza test result, no. (%)		Inpatient setting	Influenza test result, no. (%)	
		Positive	Negative		Positive	Negative
**IVY**						
≥18 yrs	—	—	—	3,175	675 (21)	2,500 (79)
**NVSN**						
<18 yrs (6 mos–17 yrs)	2,969	482 (16)	2,487 (84)	1,642	119 (7)	1,523 (93)
**U.S. Flu VE**						
<18 yrs (8 mos–17 yrs)	1,134	217 (19)	917 (81)	—	—	—
≥18 yrs	2,210	475 (21)	1,735 (79)	—	—	—
**VISION**						
<18 yrs (6 mos–17 yrs)	36,919	9,563 (26)	27,356 (74)	1,638	157 (10)	1,481 (90)
≥18 yrs	102,639	26,011 (25)	76,628 (75)	31,033	2,959 (10)	28,074 (90)

**Abbreviations:** IVY = The Investigating Respiratory Viruses in the Acutely Ill Network; NVSN = New Vaccine Surveillance Network; U.S. Flu VE = U.S. Flu Vaccine Effectiveness Network; VE = vaccine effectiveness; VISION = Virtual SARS-CoV-2, Influenza, and Other respiratory viruses Network.

* Outpatient = outpatient clinics, urgent care, and emergency departments (NVSN and U.S. Flu VE); and urgent care and emergency departments (VISION).

^†^ Patients enrolled as outpatients in NVSN might have progressed to a more acute level of care; those data might not be reflected in this analysis.

### Influenza Vaccination Status Among Control Patients

Among control patients (i.e., those patients with ARI and a negative influenza test result) aged <18 years, the percentage vaccinated ranged from 22% (VISION) to 34% (NVSN) in outpatient settings, and from 27% (VISION) to 40% (NVSN) in the inpatient setting (Table 2). Among all adult control patients, the percentage vaccinated was 34% in outpatient settings (U.S. Flu VE and VISION) and ranged from 35% (IVY) to 39% (VISION) in the inpatient setting. Among control patients aged ≥65 years, 54% (VISION) to 59% (U.S. Flu VE) in outpatient settings and 45% (IVY) to 46% (VISION) in the inpatient setting were vaccinated.

**TABLE 2:** Number and percentage of children and adolescents* aged <18 years and adults aged ≥18 years receiving seasonal influenza vaccine, number and percentage with a positive or negative influenza test result, and vaccine effectiveness,^†^ by influenza type and subtype^§^ — four vaccine effectiveness networks, United States, 2024–25 influenza season

Network (setting)	Influenza test result by influenza vaccination status, no. vaccinated/Total (%)		VE (95% CI)^¶^
	Influenza-positive	Influenza-negative	
**All ages**			
Any** influenza			
**VISION (outpatient)**	6,953/35,574 (20)	31,785/103,984 (31)	56 (54 to 58)
**U.S. Flu VE (outpatient)**	166/692 (24)	848/2,652 (32)	42 (29 to 54)
**All children and adolescents aged <18 yrs**			
Any** influenza			
NVSN^††^ (outpatient^§§^)	100/482 (21)	855/2,487 (34)	59 (47 to 68)
U.S. Flu VE (outpatient)	54/217 (25)	256/917 (28)	32 (1 to 54)
VISION (outpatient)	1,322/9,563 (14)	5,943/27,356 (22)	60 (56 to 63)
NVSN (inpatient)	28/119 (24)	613/1,523 (40)	63 (41 to 76)
VISION (inpatient)	16/157 (10)	406/1,481 (27)	78 (60 to 89)
**Influenza A(H1N1)pdm09**			
NVSN (outpatient)	32/224 (14)	855/2,487 (34)	72 (59 to 81)
U.S. Flu VE (outpatient)	9/50 (18)	256/917 (28)	53 (3 to 79)
NVSN (inpatient)	13/60 (22)	613/1,523 (40)	63 (30 to 81)
**Influenza A(H3N2)**			
NVSN (outpatient)	62/218 (28)	855/2,487 (34)	42 (19 to 58)
U.S. Flu VE (outpatient)	29/107 (27)	256/917 (28)	16 (–34 to 49)
NVSN (inpatient)	12/44 (27)	613/1,523 (40)	55 (14–77)^¶¶^
**All adults aged ≥18 yrs**			
**Any**^¶^ **influenza**			
U.S. Flu VE (outpatient**)	112/475 (24)	592/1,735 (34)	36 (16 to 51)
VISION (outpatient)	5,631/26,011 (22)	25,842/76,628 (34)	54 (52 to 56)
IVY (inpatient)	211/675 (31)	873/2,500 (35)	41 (28 to 52)
VISION (inpatient)	905/2,959 (31)	10,869/28,074 (39)	55 (51 to 59)
**Influenza A(H1N1)pdm09**			
U.S. Flu VE (outpatient)	36/118 (31)	592/1,735 (34)	42 (8 to 64)
IVY (inpatient)	12/50 (24)	873/2,500 (35)	39 (−14 to 67)
**Influenza A(H3N2)**			
U.S. Flu VE (outpatient)	56/230 (24)	592/1,735 (34)	25 (–6 to 48)
IVY (inpatient)	28/110 (26)	873/2,500 (35)	51 (22 to 69)
**Adults aged 18–64 yrs**			
**Any Influenza**			
U.S. Flu VE (outpatient)	84/419 (20)	397/1,403 (28)	37 (16 to 53)
VISION (outpatient)	3,056/20,280 (15)	10,864/49,103 (22)	56 (53 to 58)
IVY (inpatient)	61/334 (18)	282/1,187 (24)	48 (28 to 63)
VISION (inpatient)	212/1,062 (20)	1,966/8,803 (22)	51 (41 to 59)
**Adults aged ≥65 yrs**			
**Any influenza**			
U.S. Flu VE (outpatient)	28/56 (50)	195/332 (59)	18 (–69 to 60)
VISION (outpatient)	2,575/5,731 (45)	14,978/27,525 (54)	51 (47 to 54)
IVY (inpatient)	150/341 (44)	591/1,313 (45)	38 (19 to 52)
VISION (inpatient)	693/1,897 (37)	8,903/19,271 (46)	57 (52 to 61)

**Abbreviations:** IVY = The Investigating Respiratory Viruses in the Acutely Ill Network; NVSN = New Vaccine Surveillance Network; OR = odds ratio; U.S. Flu VE = U.S. Flu Vaccine Effectiveness Network; VE = vaccine effectiveness; VISION = Virtual SARS-CoV-2, Influenza, and Other respiratory viruses Network.

* Aged 6 months–17 years (NVSN and VISION); 8 months–17 years (U.S. Flu VE).

^†^ VE was estimated using the test-negative design comparing odds of receipt of 2024–2025 influenza vaccination among persons with an acute respiratory illness who received a positive influenza test result with those among persons who received a negative influenza or SARS-CoV-2 test result. ORs were estimated using logistic regression; VE was calculated as (1 − adjusted OR) × 100%. Firth logistic regression was used for estimates from IVY

^§^ Subtype was not available for VISION.

^¶^ All networks were adjusted for geographic region, age, and calendar time. IVY, U.S. Flu VE, and VISION were adjusted for sex and race and ethnicity.

** As of February 1, 2025, most influenza viruses detected have been influenza A viruses (97% of positive specimens).

^††^ Patients enrolled as outpatients in NVSN might have progressed to a more acute level of care, and those data might not be reflected in this analysis.

^§§^ Outpatient = outpatient clinics, urgent care, and emergency departments (NVSN and U.S. Flu VE); or urgent care and emergency departments (VISION).

^¶¶^ Firth logistic regression was used for this estimate.

### VE against ARI in Outpatient and Inpatient Settings

**Children and adolescents.** Among persons aged <18 years, VE against any influenza-associated ARI was 32% (U.S. Flu VE), 59% (NVSN), and 60% (VISION) in outpatient settings and 63% (NVSN) and 78% (VISION) against influenza-associated hospitalization. Against influenza A(H1N1)pdm09, VE was 72% (NVSN) and 53% (U.S. Flu VE) in outpatient settings, and 63% (NVSN) against influenza-associated hospitalization. Against influenza A(H3N2), VE was 42% (NVSN) in outpatient settings and 55% (NVSN) against influenza-associated hospitalization. The estimate of VE in outpatient settings in the U.S. Flu VE network was not statistically significant (16%; 95% CI = −34% to 49%).

**Adults.** Among persons aged ≥18 years, VE against any influenza-associated ARI was 36% (U.S. Flu VE) and 54% (VISION) in outpatient settings and 41% (IVY) and 55% (VISION) against influenza-associated hospitalization. Effectiveness against influenza A(H1N1)pdm09 was 42% in outpatient settings (U.S. Flu VE) but was not statistically significant against influenza-associated hospitalization in the IVY network (39%; 95% CI = −14% to 67%). Effectiveness against influenza A(H3N2) was 51% (IVY) against influenza-associated hospitalization but was not statistically significant in the outpatient setting (25%; 95% CI = −6% to 48%, U.S. Flu VE network).

Among adults aged 18–64 years, VE against any influenza-associated ARI in outpatient settings was 37% (U.S. Flu VE) and 56% (VISION); VE against hospitalization was 48% (IVY) and 51% (VISION). Among adults aged ≥65 years, VE against any influenza-associated ARI was 51% (VISION) in outpatient settings and was 38% (IVY) and 57% (VISION) against hospitalization; VE was not statistically significant in the outpatient setting in the U.S. Flu VE network (VE = 18%; 95% CI = −69% to 60%).

### Genetic Characterization of Influenza Viruses

As of February 3, 2025, a total of 286 influenza A(H3N2) viruses were genetically characterized, including 26 (9%) from patients in IVY, 200 (70%) from the U.S. Flu VE network, and 60 (21%) from NVSN; all belonged to the hemagglutinin (HA) clade 2a.3a.1, which includes the A(H3N2) strain selected for the 2024–2025 cell-grown influenza vaccine (A/Massachusetts/18/2022) (Supplementary Table 2, https://stacks.cdc.gov/view/cdc/176590) Among 158 sequenced A(H1N1)pdm09 viruses, five (3%) were from IVY, 80 (51%) were from the U.S. Flu VE network, and 73 (46%) were from NVSN. Among these, three from IVY, 55 from U.S. Flu VE, and 46 from NVSN belonged to HA clade 5a.2a, and two from IVY, 25 from U.S. Flu VE, and 27 from NVSN belonged to HA clade 5a.2a.1. The HA clade 5a.2a.1 includes the A(H1N1)pdm09 strain selected for the 2024–2025 cell-grown influenza vaccine (A/Wisconsin/67/2022).

## Discussion

These interim estimates of 2024–25 VE indicate that influenza vaccination was effective in preventing medically attended influenza-associated illness in children, adolescents, and adults in the United States. Among children and adolescents, VE against medically attended influenza ranged from 32% to 60% in outpatient settings and from 63% to 78% against influenza-associated hospitalization. Among adults, VE against medically attended influenza was 36% and 54% in two outpatient settings and 41% and 55% against influenza-associated hospitalization. Despite increased circulation of influenza A(H3N2) viruses, which are generally associated with lower VE (*3*), estimates from this influenza season were consistent with those from the 2023–24 season and seasons associated with higher VE over the last 15 years (*4*). These estimates are also similar to interim estimates from Canada for the 2024–25 influenza season, which estimated VE to be 54% overall (*5*), and estimates from South America for the 2024 southern hemisphere influenza vaccine, which estimated VE against influenza A to be 34% overall (*6*). Given the high levels of influenza activity and severity in the United States this season, increasing influenza vaccination could reduce influenza-associated illnesses, medical visits, hospitalizations and deaths.^¶¶¶^

The VE estimates and associated confidence levels included in this report might reflect regional variations in circulating viruses. In U.S. Flu VE, most subtyped influenza A specimens (67%) were influenza A(H3N2) compared with 48% in NVSN. The U.S. Flu VE network did not find statistically significant VE against influenza A(H3N2) in the outpatient setting among child and adolescent patients or among adult patients. The VE estimates against influenza A(H3N2) are similar to findings from the 2018–19 season (*7*) and to findings from Europe during the 2024–25 influenza season (*8*). To address evolutionary changes in the influenza virus, the composition of influenza vaccines is reviewed annually; influenza vaccines are updated to protect against the influenza viruses that data indicate are most likely to be circulating during the following influenza season. When circulating viruses are antigenically different from the vaccine viruses, influenza VE can be reduced.****

### Limitations

The findings in this report are subject to at least four limitations. First, these VE estimates are preliminary, and end-of-season estimates might be different as influenza continues to spread during the 2024–25 season. Second, influenza vaccination status might be misclassified in some networks, which could affect VE estimates. Vaccines administered in pharmacies are routinely reported to jurisdictional immunization information systems (IISs), although vaccination clinics conducted in nontraditional settings such as workplaces might not be reported to IISs. Third, patients who had received ≥1 dose of the 2024–2025 influenza vaccine were considered vaccinated; however, children aged 6 months–8 years are recommended to receive 2 doses if they have not previously received ≥2 doses. Therefore, some children who were classified as vaccinated might not have been fully vaccinated, which could reduce VE estimates. Finally, the potential for unmeasured confounding exists, because networks did not control for variables such as previous vaccination, previous influenza virus infection, or underlying medical conditions. 

### Implications for Public Health Practice

Vaccination is the best way to prevent influenza and influenza-associated hospitalization. Findings in this report show that vaccination with the 2024–2025 influenza vaccine reduced the likelihood of medically attended influenza and support CDC’s recommendation that all persons aged ≥6 months be vaccinated against influenza (*1*). These findings also support the strong protective effect influenza vaccination has against influenza-associated hospitalization, demonstrating the importance of vaccination to reduce more severe influenza-associated complications. Eligible persons aged ≥6 months who have not received the 2024–2025 influenza vaccine should get vaccinated as long as influenza viruses circulate locally.

## Footnotes

*
https://www.cdc.gov/flu-vaccines-work/php/effectiveness-studies/index.html

†
https://www.cdc.gov/flu-burden/php/data-vis-vac/index.html

§
https://www.fda.gov/vaccines-blood-biologics/lot-release/use-trivalent-influenza-vaccines-2024-2025-us-influenza-season

¶
https://www.cdc.gov/fluview/surveillance/2025-week-05.html

** To reduce potential case misclassification, all influenza case-patients received a positive reverse transcription–polymerase chain reaction test result from a clinical or surveillance respiratory laboratory specimen for IVY, NVSN, and U.S. Flu VE. For VISION, influenza case-patients received a positive molecular assay result from a clinical respiratory laboratory specimen.

†† Patients enrolled as outpatients in NVSN might have progressed to a more acute level of care, and those data might not be reflected in this analysis.

§§ IVY, U.S. Flu VE, and VISION also adjusted for sex and race and ethnicity.

¶¶ IVY, NVSN, and U.S. Flu VE used date of ARI onset. VISION used the earlier of outpatient visit date, hospital admission date, or influenza clinical testing date.

*** VISION also excluded patients who received a negative influenza test but a clinical diagnosis of influenza, patients who received a clinical diagnosis of COVID-19, and influenza case-patients who received a positive molecular test for respiratory syncytial virus.

††† 45 C.F.R. part 46.102(l)(2), 21 C.F.R. part 56; 42 U.S.C. Sect. 241(d); 5 U.S.C. Sect. 552a; 44 U.S.C. Sect. 3501 et seq.

§§§ 45 C.F.R. part 46, 21 C.F.R. part 56; 42 U.S.C. Sect. 241(d); 5 U.S.C. Sect. 552a; 44 U.S.C. Sect. 3501 et seq.

¶¶¶
https://www.cdc.gov/flu-burden/php/data-vis/2024-2025.html

****
https://www.cdc.gov/flu/vaccines/keyfacts.html
